# Recent advances in the detection of base modifications using the Nanopore sequencer

**DOI:** 10.1038/s10038-019-0679-0

**Published:** 2019-10-11

**Authors:** Liu Xu, Masahide Seki

**Affiliations:** Department of Computational Biology and Medical Sciences, Graduate School of Frontier Sciences, The University of Tokyo, Kashiwa, Chiba Japan

**Keywords:** RNA sequencing, DNA sequencing

## Abstract

DNA and RNA modifications have important functions, including the regulation of gene expression. Existing methods based on short-read sequencing for the detection of modifications show difficulty in determining the modification patterns of single chromosomes or an entire transcript sequence. Furthermore, the kinds of modifications for which detection methods are available are very limited. The Nanopore sequencer is a single-molecule, long-read sequencer that can directly sequence RNA as well as DNA. Moreover, the Nanopore sequencer detects modifications on long DNA and RNA molecules. In this review, we mainly focus on base modification detection in the DNA and RNA of mammals using the Nanopore sequencer. We summarize current studies of modifications using the Nanopore sequencer, detection tools using statistical tests or machine learning, and applications of this technology, such as analyses of open chromatin, DNA replication, and RNA metabolism.

## Introduction

### DNA and RNA modifications

More than 17 and 160 types of modified bases have been found in DNA and RNA, respectively [1, 2]. DNA modification plays roles in several biological processes, including development, aging, and cancer [3–5]. In vertebrates, m5C (5-methylcytosine) is frequently observed in CpG dinucleotides in DNA [3]. The methylation of cytosine is catalyzed by DNMTs (DNA methyltransferases). By methylating the CpGs of the newly synthesized strand complementary to methylated CpG, DNMT1 maintains the methylation of cytosine beyond DNA replication. DNMT3A and B are responsible for de novo methylation. CpG islands, where there is a high frequency of CpGs are predominately found in promoter regions. m5C at CpG islands in the promoters represses gene expression [3]. m5C is also important for the repression of transposable elements [6]. MBDs (Methyl-CpG-binding domain proteins) bind to methylated CpG sites, and recruit histone-modifying enzymes and chromatin remodeling complexes to organize the repressive chromatin structure [7]. hm5C (5-hydroxymethylcytosine) is generated by the oxidation of 5mC catalyzed by TET (Ten–eleven translocation) enzymes [8]. hm5C is predominantly observed in embryonic stem cells and neurons. hm5C is known as an intermediate in demethylation via the TET–TDG pathway. Although the underlying mechanism remains elusive, it is suggested that the function of hm5C is important for the maintenance of pluripotency and alternative splicing.

RNA modifications are also important for biological processes including development and cancer [9, 10]. Noncoding RNA, such as ribosomal RNA and transfer RNA, harbor several types of RNA modifications, and these modifications are indispensable for their proper function [11]. m6A (N6-methyladenine) is the most common modification found in the mRNA of eukaryotes and is catalyzed by METTL3, METTL14, and WTAP [11]. m6A is recognized by m6A-binding proteins, such as YTHDC1 and YTHDF3, and regulates mRNA processing, stability, translation, and transportation [12–15]. A-to-I (adenine to inosine) editing is also frequent in the RNA of higher eukaryotes [11]. A-to-I editing is catalyzed by ADAR (adenosine deaminase acting on RNA) enzymes. Due to the base pairing of inosine with cytosine, inosine is recognized as guanosine during splicing and translation. Inosine is converted to guanosine via cDNA synthesis and is detected as A-to-G mismatch through sequencing.

### Nanopore sequencing

The Nanopore sequencer released by Oxford Nanopore Technologies, performs DNA/RNA sequencing directly and in real time. The Nanopore sequencer carries out sequencing by predicting sequences from electric current patterns knows as “squiggle”, which are affected by the bases inside the Nanopore. Oxford Nanopore Technologies has released several Nanopore sequencing instruments, including the MinION and PromethION sequencers [16, 17]. The MinION is a palmtop-sized sequencer. Taking advantage of the portability and real-time sequencing ability of MinION, this system has been applied in various situations, including field research and clinical diagnosis [18, 19]. The MinION flowcell has 512 pores in which sequencing can be performed at the same time [15]. The maximum throughput of MinION is 10–30 Gb per single MinION flowcell based on the manufacturer’s data. In a previous human genome study by our group, MinION stably generated over 3 Gb of DNA sequences in a single run [20]. The maximum length of its reads is greater than 2 Mb [21]. PromethION is a high-throughput stationary sequencer. PromethION can run up to 48 flowcells in parallel [16]. The PromethION flowcell has 3000 pores in which sequencing can be performed at the same time. For DNA sequencing, 158 Gb of data can be generated per single PromethION flowcell based on the manufacturer’s data [16]. Therefore, PromethION can generate 7.6 Tb of sequence data. The average read length of human genome sequences without size selection is more than 10 kb for both the MinION and PromethION [20]. Although the throughput is becoming comparable with that of short-read sequencing, the accuracy of Nanopore sequencing (83–95%, depending on the library type) is lower than that of short-read sequencing using the Illumina platforms (99.9%) [22, 23].

**Fig. 1:**
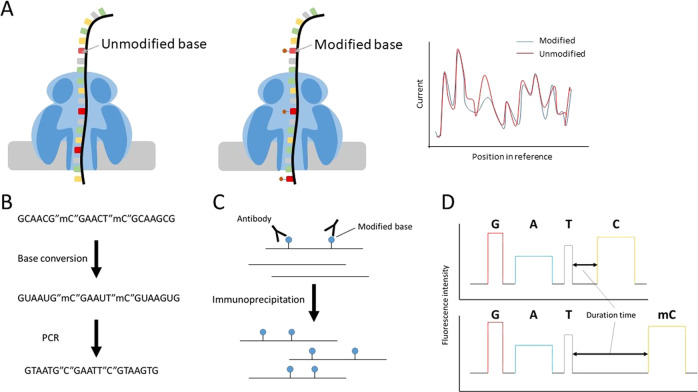
Modified base detection using Nanopore sequencing and general methods. Schema of modified base detection using the Nanopore sequencer (**a**) and through bisulfite conversion (**b**), immunoprecipitation of nucleic acids (**c**), and SMRT sequencing (**d**)

Existing short-read sequencing-based methods for modified base detection have difficulty in determining the modification patterns of a chromosome or transcript and cannot detect modifications without an immunoprecipitation-grade antibody, distinguishable base conversion method, or the introduction of reverse transcription errors by modified bases [2, 24]. These detection methods are available for very limited kinds of modified bases. The Nanopore sequencer can read native single DNA and RNA molecules harboring base modifications without PCR amplification or cDNA conversion [25–27]. Nanopore sequencing can detect modified bases according to differences in squiggles between modified and unmodified base (Fig. 1a). The Nanopore sequencer is applied to detect several kinds of modified bases, such as m5C and m6A in DNA and m6A in RNA, in long single nucleic acid molecules [25, 26]. Modified bases can be detected based on significantly unique squiggle patterns. For the detection of modifications, some tools in which machine learning and statistical tests are applied are available at present. Recently, application methods have been developed in addition to simply detecting modifications. Here, we describe recent advances in the detection of base modifications using the Nanopore sequencer.

## General methods for base modification detection

### Methods using short-read sequencing

For the detection of m5C and hm5C on DNA, BS-seq (bisulfite sequencing) approaches such as WGBS (whole genome bisulfite sequencing) and RRBS (reduced representation bisulfite sequencing), based on bisulfite conversion and subsequent short-read sequencing, are the most commonly applied methods [28] (Fig. 1b). While the whole genome is analyzed by WGBS, CpG-rich regions are mainly analyzed by RRBS [29]. In RRBS, a short-read sequencer preferentially reads CpG-rich short fragments generated by digestion using the restriction enzyme MspI, which recognizes the “CCGG” sequence motif. To discriminate m5C and hm5C from umC (unmethylated cytosine), umC is converted to uracil via bisulfite conversion, and uracils are replaced with thymines in the subsequent PCR step. Although BS-seq is unable to discriminate between m5C and hm5C, other bisulfite conversion-based methods, such as oxBS-seq (oxidative bisulfite sequencing) or TAB-seq (Tet-assisted bisulfite sequencing), are able to detect only m5C or hm5C, respectively [30, 31]. Bisulfite conversion is also used to detect m5C on RNA [32]. Because bisulfite conversion is a chemically severe reaction, a substantial proportion of nucleic acids are fragmented, and analyzing the methylation patterns of long nucleotides by combining this method and long-read sequencing is therefore difficult [33]. Recently, nondestructive methods for base conversion other than bisulfite conversion have been reported by some groups [34–36]. The EM-seq (enzymatic methyl-seq) method has been released by New England Biolabs, Inc., and ACE-seq (APOBEC-coupled epigenetic sequencing) employs the cytidine deaminase APOBEC. EM-seq is a detection method for m5C and hm5C [34]. After the oxidation of m5C and hm5C to ca5C (5-carboxylcytosine) for protection from deaminase, umC is converted to uracil by APOBEC. Similar to BS-seq, umC is sequenced as a T base. ACE-seq is a detection method for hm5C [35]. After the glucosylation of hm5C for protection, m5C and unmethylated cytosines are converted to uracil by APOBEC3A. In TAPS (TET-assisted pyridine borane sequencing), after the oxidation of m5C and hm5C to ca5C, ca5C is converted to dihydrouracil via pyridine borane reduction [36]. Therefore, in contrast to BS-seq and EM-seq, m5C and hm5C are sequenced as T bases in TAPS. Longer DNA can be obtained using these methods than by bisulfite conversion. Although it is unclear whether uracil or dihydrouracil can be sequenced by long-read sequencers, these methods may be applicable for long-read sequencing.

For the detection of m6A, methods based on the short-read sequencing of RIP (RNA immunoprecipitation) samples, such as MeRIP-seq (methylated RIP sequencing) and m6A-seq, are the most prevalent [37, 38] (Fig. 1c). RIP-based methods rely on the specificity and affinity of an antibody that recognizes m6A. However, MeRIP-seq and m6A-seq are unable to reveal the specific position of the m6A. miCLIP (m6A individual nucleotide resolution crosslinking and immunoprecipitation) can identify the specific m6A position [39]. miCLIP involves the UV-crosslinking of an anti-m6A antibody to m6A sites and truncation or misincorporation of C bases at m6A sites during reverse transcription caused by crosslinking. This method requires short fragmented RNA as the input. Therefore, accurate estimation of expression and the full-length structure of RNA with m6A is difficult. In m6A-LAIC-seq (m6A-level and isoform-characterization sequencing), unfragmented RNA is used as the input for immunoprecipitation [40]. This method can detect structural isoforms of transcripts similar to typical RNA-seq. For the detection of DNA modifications, immunoprecipitation-based approaches, such as methylated DNA immunoprecipitation sequencing for m5C, are also used [41]. However, immunoprecipitation-based methods are limited by bias based on the immunoprecipitation efficiency.

### SMRT (single molecule, real-time) sequencing

SMRT sequencing with long-read sequencers released by Pacific Bioscience can detect DNA modifications. In the PacBio Sequel sequencer, SMRT cell harboring a million wells is used for SMRT sequencing. The complex of a DNA polymerase and a single DNA molecule is fixed to each well of SMRT cell. SMRT sequencing is performed by continuously observing the incorporation of a fluorescently labeled nucleotide by DNA polymerase in each well. Approximately 365,000 reads with an average length of 10–14 kb are generated by the PacBio Sequel sequencer per an SMRT cell [42]. On the basis of differences in the duration of nucleotide incorporation between unmodified and modified bases, SMRT sequencing can detect the positions and kinds of base modifications (Fig. 1d). SMRT sequencing has been applied to detect various DNA modifications, such as m5C, m6A, and hm5C [43]. The detectability of a modification depends on the magnitude of the effect of the modification on the kinetics of the polymerase. Because the methyl group of m5C does not directly contribute to base pairing, m5C shows a subtler effect on nucleotide incorporation than m6A [44]. Therefore, relatively high-coverage sequencing or oxidation of m5C to ca5C using TET1 for signal enhancement is required for the detection of m5C [45]. Minimum 25X and 250X coverage are recommended for the detection of m6A and m5C [46]. Although 6 mA in DNA is responsible for biological functions in bacteria, its function is unclear and it is very rare in metazoan [47]. It is suggested that SMRT sequencing overestimates 6 mA in DNA in which m6A is rare. SMRT-BS (SMRT sequencing of bisulfite-converted DNA) using gene-specific primers has also been developed [48]. However, the read length of SMRT-BS is up to ~1.5 kbp and SMRT-BS is not applied for genome-wide sequencing. For RNA, Iso-Seq, which is a SMRT sequencing method for full-length cDNA, can be used for the determination of entire sequences of transcript isoforms. However, due to the requirement for full-length cDNA synthesis, RNA modification information is lost. SMRT sequencing is also compatible with direct sequencing of RNA molecules using reverse transcription [49]. Vilfan et al. used this approach for the detection of m6A and RNA secondary structure. However, the detection of RNA modifications using SMRT sequencing has only rarely been performed in other studies.

## Modified base detection using the Nanopore sequencer

### Genome sequencing

Whole-genome sequencing and methylation calling using Nanopore sequencing has been performed in mammals, including humans and mice [26, 50, 51]. Jain et al. conducted whole-genome sequencing of a human cell line at approximately 30X coverage using MinION [50]. These authors compared CpG methylation frequencies on chromosome 20 called from Nanopore sequencing data using *Nanopolish* and short-read-based BS-seq. The data showed high correlations, with *r* = 0.895. Gigante et al. conducted whole-genome sequencing of F1 B6 and Cast strain hybrid mice with ~10X coverage using MinION and PromethION [51]. They called the CpG methylation status using *Nanopolish* and compared the data with those obtained from RRBS with an Illumina sequencer. In addition, the authors conducted haplotyping of the Nanopore and Illumina reads utilizing SNPs between the B6 and Cast strains and showed that a larger fraction of the Nanopore reads were assigned compared with the Illumina reads. Known and novel imprinting control regions were detected by Nanopore sequencing.

To analyze the modification of target regions without performing whole-genome sequencing, native DNA must be enriched without PCR. Methods involving Cas9-mediated enrichment have been developed to enrich a target region [52, 53]. Gilpatrick et al. developed nCATS (nanopore Cas9-targeted sequencing), which is a simple target enrichment method [52]. In nCATS, all of the 5′ ends of the DNA are dephosphorylated to prevent adapter ligation to unwanted DNA. After dephosphorylation, the Cas9 enzyme and a guide RNA designed for both ends of the target region are applied to cut the target region. The Nanopore sequencing adapters are specifically ligated to the 5′ phosphate of the target DNA generated by Cas9. Gilpatrick et al. applied nCAT to ten loci of ~18 kb in length from three human cell lines and obtained a median coverage of 165X at the ten loci from a MinION flowcell. They used the enriched reads for CpG methylation calling and the detection of structural variants.

### Direct RNA-seq

Nanopore sequencing enables the direct sequencing of RNA molecules, which is referred to as direct RNA-seq [25]. Direct RNA-seq reveal full-length RNA structures and modifications simultaneously. Because direct RNA-seq is a PCR and reverse transcription-free method, it shows less bias than short-read-based RNA-seq and Nanopore cDNA-seq, which use reverse transcription and PCR amplification of cDNA. Garalde et al. showed differences in the squiggles associated with m6A and m5C compared with those of unmodified bases using synthetic RNA [25]. Smith et al. conducted direct RNA-seq of 16S rRNA in a wild type *Escherichia coli* strain, two mutant strains lacking a guanine methyltransferase or a pseudouridine synthases and a strain expressing another guanine methyltransferase [54]. They indicated that alternation of current signals and base calling error were observed around position where 7-methylguanosine and pseudouridine occurred. Workman et al. performed direct RNA-seq analysis of RNA from a human cell line, in vitro transcribed RNA from cDNA from the same cell line, and synthetic RNA [55] with a focus on the m6A methyltransferase-binding motif. They detected current differences for the motif and validated the differences in signals using data from the synthetic RNA. Utilizing the current difference, they detected m6A-modified motif in 57 genes. Furthermore, they attempted to detect A-to-I editing. They showed systematic miscalling and a change in the ion current for direct RNA-seq reads and A-to-G mismatch in cDNA-seq reads at A-to-I sites. They also measured polyA tail length using the polya option of *Nanopolish* and showed differences in polyA tail length both between genes and between transcripts of the same gene. Finally, Viehweger et al. performed direct RNA-seq analysis of human coronavirus and detected m5C in the viral RNA using *Tombo* [56, 57].

## Tools for modified base detection

**Fig. 2:**
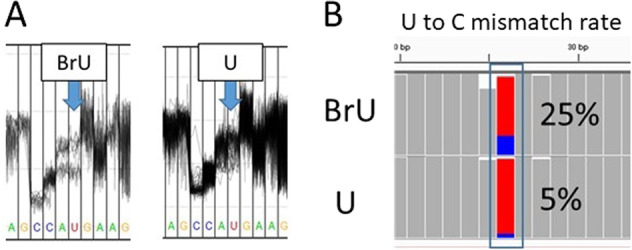
Tools for modified base detection using Nanopore sequencing. Differences in the squiggles (**a**) and mismatch rates (**b**) of an artificial bromouridine base are shown. The direct RNA-seq reads of synthetic RNA were aligned using minimap2 [58]. The squiggle plot was drawn using Tombo [57]. The aligned reads were visualized using IGV [79]

Tools for the detection of modifications based on several principles have been released (Table 1). In most tools except for the base calling-based tools, the reads must first be aligned to the reference sequence using alignment software, such as *minimap2* [58]. For tools utilizing current signals, the current intensity is associated with the aligned base position using tools such as resquiggle in *Tombo* and eventalign in *Nanopolish* [26, 57] (Fig. 2a). A modified base is detected based on comparison with the training model or the squiggle of an unmodified nucleic acid. *Nanopolish*, which is based on the HMM (hidden Markov model), can call m5Cs on DNA in the CpG context [26]. *Nanopolish* is widely used for m5C calling in various species, including humans [50]. *SignalAlign* is based on the HMM with the hierarchical Dirichlet process [27]. Rand et al. detected m5C, hm5C, and m6A in *E. coli* DNA using *SignalAlign*. They showed that the detection of m5C was easier than that of m6A, contrary to the situation in SMRT sequencing. *mCaller* can use the four machine learning classifiers (neural network, random forest, logistic regression, and naive Bayes classifiers) to detect m6A on DNA [59]. Mclntyre et al. showed that the predictor using the neural network was the most accurate. They also compared the detection of m6A on DNA using *mCaller* and *Tombo*. They showed that which tool is better depends on the sequence motif. *DeepSignal* and *DeepMod* detect m5C and m6A in DNA using neural networks [60, 61]. Ni et al. compared *DeepSignal* with *Nanopolish* and *SignalAlign* for the detection of m5C or m5C and m6A, respectively [60]. *DeepSignal* showed a higher performance, in terms of both accuracy and sensitivity than the other two tools. *DeepSignal* can detect m5C at CpGs with ~0.92 accuracy at 1X coverage and m6A at GATC motif with ~0.90 accuracy at 2X coverage. *DeepMod* showed precision up to 0.99 and 0.9 for m5C and m6A, respectively [61]. In the detection of m6A, *DeepMod* showed slightly higher performance than *mCaller*. Statistical test-based tools can detect de novo modifications without training using modified and unmodified samples. *Nanoraw* employs the Mann–Whitney U test [57]. *Tombo* is the successor of *Nanoraw* and supports the detection of m5C on DNA and RNA and m6A on DNA in all sequence contexts without a requirement for unmodified samples in addition to de novo detection. *NanoMod* uses the Kolmogorov–Smirnov test [62]. Liu et al. showed that *NanoMod* presented higher performance than *Nanoraw* for the detection of m5C in *E. coli*.

**Table 1:** Tools for modified base detection

Tool	Target	URL
Nanopolish	DNA	https://nanopolish.readthedocs.io/en/latest/index.html
SignalAlign	DNA	https://github.com/ArtRand/signalAlign
mCaller	DNA	https://github.com/al-mcintyre/mCaller
DeepSignal	DNA	https://github.com/bioinfomaticsCSU/deepsignal
DeepMod	DNA	https://github.com/WGLab/DeepMod
NanoMod	DNA	https://github.com/WGLab/NanoMod
Tombo	DNA/RNA	https://github.com/nanoporetech/tombo
ELIGOS	RNA	https://bitbucket.org/piroonj/eligos.git
EpiNano	RNA	https://github.com/enovoa/EpiNano/issues
Flappy	DNA	https://github.com/nanoporetech/flappie
Taiyaki	DNA	https://github.com/nanoporetech/taiyaki

Due to differences in the current signal between modified and unmodified bases, there is a tendency for higher error rates to be observed at modified than at unmodified bases (Fig. 2b). Wongsurawat et al. developed *ELIGOS* (epitranscriptional landscape inferring from glitches of ONT signals), which is a tool for the detection of RNA modifications [63]. *ELIGOS* detects RNA modifications by comparing the % error at a specific base between direct RNA-seq analyses of modified and unmodified RNA or direct cDNA-seq analysis, in which the modification information is erased during reverse transcription. They validated *ELIGOS* through the detection of known modifications in *Saccharomyces cerevisiae*, *E. coli*, and human cell line ribosomal RNA. The authors applied *ELIGOS* for the detection of modification from *S. cerevisiae* transcriptome data and to the detection of m6A and G-quadruplex (a type of secondary structure) from human cell line transcriptome data. *EpiNano* is a tool for the detection of the m6A modification of RNA [64]. *EpiNano* distinguishes modified bases by support vector classification using quality scores, mismatch patterns of base-called sequences, and raw current signals as input data. *EpiNano* detect the m6A modification in vivo with an accuracy of 0.87. Although *ELIGOS* and *EpiNano* can detect sites with modifications from bulk reads, they are not compatible with detection of modified bases in single reads at present.

Although the tools described thus far require base-called sequence data, some base callers can base call modified bases directly. *Flappie*, which was released by Oxford Nanopore Technologies, is a base caller based on the ‘flip-flop’ algorithm [65]. m5C in the CpG context of DNA is called as the an extra base in addition to the four standard bases by *Flappie*. *Taiyaki* is a model training tool for the Nanopore base caller that can be used in the model training of modified bases [66]. The model trained on *Taiyaki* is usable in *Guppy*, which is a Nanopore base caller. These base callers calling modified bases have not been benchmarked thus far.

As described above, many detection tools for modified bases have become available recently. In the detection of m5C and m6A on DNA, the tools based on neural networks tend to show higher performance than other tools [60, 61]. For the detection of de novo modifications, statistical test-based tools is applicable [57, 62]. Because comparison results are highly dependent on comparison conditions, such as the sequence context and datasets, fair comparison by a third party is necessary for accurate assessment.

## Application of modified base detection by the Nanopore sequencer

### Analysis of open chromatin regions

**Fig. 3:**
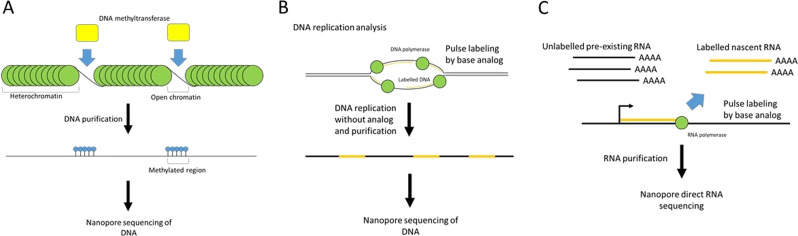
Applied methods for modified base detection using Nanopore sequencing. **a** Detection of open chromatin regions using DNA methyltransferase accessibility. DNA in open chromatin regions is specifically methylated. Open chromatin regions are detected as highly methylated regions by Nanopore sequencing. **b** Detection of DNA replications using base analogs. The replicated regions are labeled by pulse labeling during S phase. The replicated regions are detected as labeled regions by Nanopore sequencing. **c** Detection of nascent RNA using base analogs. Nascent RNAs are labeled by pulse labeling using base analogs. Nascent and preexisting RNAs are classified by the existence of base analogs

DNase-seq, ATAC-seq, and NOMe-seq are short-read sequencing-based methods for the detection of open chromatin regions not protected by nucleosomes. Whereas DNase-seq and ATAC-seq utilize the accessibility of a DNase or transposase to open chromatin and DNA fragmentation by these enzymes, NOMe-seq utilizes the accessibility of a GpC-specific methyltransferase (M.CviPI) to open chromatin and detects open chromatin regions as methylated GpC regions, using BS-seq [67]. nanoNOMe, MeSMLR-seq, and SMAC-seq are methods that combine the accessibility of one or more methyltransferases and the direct detection of methylated regions by the Nanopore sequencer instead of requiring BS-seq [68–70]. These methods can detect open chromatin patterns of single long DNA molecules (Fig. 3a). Although nanoNOMe and MeSMLR-seq employ a GpC methyltransferase similar to NOMe-seq, SMAC-seq employs a m6A methyltransferase (EcoGII) and CpG-specific methyltransferase (M.SssI) in addition to the GpC methyltransferase to increase the resolution [68]. Shipony et al. and Wang et al. applied SMAC-seq and MeSMLR-seq to S. cerevisiae, respectively [68, 70]. In SMAC-seq, *Tombo* is used for methylation detection [68]. Lee et al. applied nanoNOMe to four human cell lines and detected DNA methylation using *Nanopolish* [69]. Furthermore, they investigated not only open chromatin patterns but also CpG methylation from single reads and showed an anticorrelation between chromatin accessibility and DNA methylation.

### Detection of DNA replication using base analogs

For genome-wide analysis of DNA replication, pulse labeling of replication sites by base analogs, such as BrdU (bromodeoxyuridine), is widely used [71]. Attempts to detect base analogs directly using a Nanopore sequencer have been reported by some groups [72–74] (Fig. 3b). Müllar et al. developed D-Nascent (detecting nucleotide analog signal currents on extremely long Nanopore traces) using pulse labeling by BrdU with relatively low toxicity [72]. They applied D-Nascent to analyze replication fork dynamism in *S. cerevisiae* and achieved the detection of replication sites in reads 20–160 kb in length. They also developed a BrU detection tool based on the HMM for D-Nascent. Hennion et al. used BrU for the detection of DNA replication in S. cerevisiae [73]. They developed RepNano, which is a deep learning-based software, for BrU detection. However, *RepNano* is not available at present. Georgieva et al. compared the differences in signals between thymidine and 11 species of thymidine analogs containing BrdU [74]. To detect base analogs in mouse embryonic stem cells, they employed IdU (iododeoxyuridine), which showed the greatest signal difference within a 5–6 bp k-mer used for base calling in Nanopore sequencing. They employed *NanoMod* for IdU detection.

### Measurement of RNA metabolism using base analogs

RNA abundance measured via typical transcriptome analysis is the result of RNA synthesis and degradation together. Therefore, to understand the regulatory mechanism of gene expression, RNA synthesis and degradation must be measured. Measurements of RNA synthesis and degradation can be conducted through the metabolic labeling with RNA by base analogs, as in the GRO-seq, BRIC-seq, and SLAM-seq approaches [75–77]. Because these methods are based on the sequencing of short cDNA fragments using a short-read sequencer, determining the entire transcript structure is difficult. Combining RNA labeling with direct RNA-seq using the Nanopore sequencer, Maier et al. developed Nano-ID (nanopore sequencing-based isoform dynamics), which is a method for the measurement of RNA metabolism [78] (Fig. 3c). In Nano-ID, 5-EU (5- ethynyl uridine)-labeled newly synthesized RNA is detected by direct RNA-seq. The reads derived from labeled and unlabeled RNA are distinguished using a neural network. These authors conducted Nano-ID in a human cell line and identified labeled newly synthesized isoforms and unlabeled preexisting isoforms. Furthermore, they investigated RNA isoform metabolism following heat shock stress and the correlation between the polyA length and stability.

## Conclusion

In this review, we summarized the approaches available for modified base detection using Nanopore sequencing. The emergence of Nanopore sequencers has enabled genome-wide and transcriptome-wide analyses of base modifications, including modifications for which detection methods are not available. For the detection of m6A and m5C of DNA, although high-coverage sequencing is required in SMRT sequencing, detection with a high accuracy is possible using low-coverage reads in nanopore sequencing. Many tools for the detection of modifications from Nanopore sequencing data have been developed. The systematic comparison of these tools is not sufficient. The accumulation of data on modified bases from Nanopore sequencing and the further development and maturation of the associated software will allow the detection of various modifications simultaneously. Applications involving labeling with artificial bases or artificial modification are being developed. These methods reveal the relationships of natural modifications and signatures of labeled nucleotides. Nanopore sequencing will reveal detailed regulatory mechanisms via epigenome and epitranscriptome analyses.
